# Human parvovirus B19-induced aplastic crisis in an adult patient with hereditary spherocytosis: a case report and review of the literature

**DOI:** 10.1186/1756-0500-7-137

**Published:** 2014-03-11

**Authors:** Yujin Kobayashi, Yoshihiro Hatta, Yusaku Ishiwatari, Hitoshi Kanno, Masami Takei

**Affiliations:** 1 Department of Hematology and Rheumatology, Nihon University School of Medicine, 30-1 Oyaguchi-Kamicho, Itabashi-ku, Tokyo 173-8610, Japan; 2 Division of Internal Medicine, Itabashi Medical Association Hospital, 3-12-6 Takashimadaira, Itabashi-ku, Tokyo 175-0082, Japan; 3 Department of Transfusion Medicine and Cell Processing, Tokyo Women’s Medical University, 8-1 Kawada-cho, Shinjuku-ku, Tokyo 162-8666, Japan

**Keywords:** Hereditary spherocytosis, Human parvovirus B19, Aplastic crisis

## Abstract

**Background:**

Although there are several case reports of human parvovirus B19 infection in patients with hereditary spherocytosis, no systematic reviews of adult patients with hereditary spherocytosis with human parvovirus B19 infection have been published as clinical case reports. In this study, we report a case of aplastic crisis due to human parvovirus B19 infection in an adult patient with hereditary spherocytosis.

**Case presentation:**

A 33-year-old woman with hereditary spherocytosis and gallstones was admitted because of rapid progress in marked anemia and fever. Although empiric antibiotic therapy was prescribed, her clinical symptoms and liver function test worsened. Because the anti-human parvovirus B19 antibody and deoxyribonucleic acid levels assessed by polymerase chain reaction were positive, the patient was diagnosed with aplastic crisis due to the human parvovirus B19 infection.

**Conclusion:**

We collected and reviewed several case reports of patients with hereditary spherocytosis aged > 18 years with human parvovirus B19 infection between 1984 and 2010. A total of 19 reports with 22 cases [median age, 28 years (range, 18–43 range); male: female ratio, 6:16], including the present case were identified. The male-to-female ratio of 6:16 implied that younger females were predominantly affected. Although fever and abdominal symptoms were common initial symptoms, liver dysfunction or skin eruptions were less commonly documented. Anti-human parvovirus B19 antibody or deoxyribonucleic acid levels assessed by polymerase chain reaction was commonly used to diagnose human parvovirus B19 infection and may be useful to distinguish human parvovirus B19 infection from other abdominal infection in patients with hereditary spherocytosis.

## Background

Human parvovirus (HPV)-B19 infection can cause aplastic crisis in a patient with hereditary spherocytosis (HS) associated with chronic hemolysis [1]. Although there are several case reports of HPV-B19 infection in patients with HS, particularly in children, no reports have reviewed this infection in a series of adult patients. In this study, we report a case of HPV-B19 infection-induced aplastic crisis in an adult patient. In addition to this case, we reviewed several adult patients with HPV-B19 infection and HS.

## Case presentation

**Figure 1:**
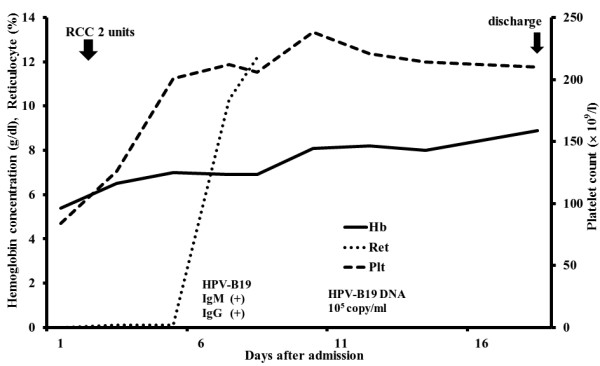
**Clinical course of the patient.** Abbreviations; RCC- Red Cell Concentrates, HPV-human parvovirus, Hb- hemoglobin, Ret- reticulocyte, Plt- platelet.

A 33-year-old woman was transferred to our hospital because of fever, general fatigue, nausea, and progressive anemia. The patient’s condition was normal until 1 week before admission, when she experienced flu-like symptoms such as fever, general fatigue, and abdominal discomfort. The patient was diagnosed with HS at the age of 6 in another hospital by the presence of hemolytic anemia, spherocytosis, increased fragility of spherocytes by osmotic fragility testing, and the absence of antibodies by direct or indirect Coombs test. Asymptomatic gallstones were diagnosed at the age of 19. The patient had undergone her annual blood test examination, and her hemoglobin concentration was maintained at approximately 10–12 g/dl. The patient was not under routine medications. Neither her parents nor her siblings had a history of HS. On admission, vital signs were as follows: blood pressure, 108/56 mmHg; pulse rate, 100 beats/min; body temperature, 39.0°C; and respiration rate, 12 breaths/min. While breathing ambient air, the patient’s oxygen saturation rate was 100%. On examination, she was alert, the skin and conjunctivae were pale, and the enlarged spleen was palpable from the costal margin. No skin rash or lymphadenopathy was observed; other physical findings were normal. The results of laboratory tests were as follows: white blood cell count was 2.97 × 10^9^/l (granulocytes, 35%; lymphocytes, 44%; atypical lymphocytes, 9%; and monocytes, 12%), red blood cell count was 1.68 × 10^12^/l; hemoglobin concentration was 5.4 g/dl; hematocrit was 14.4%; mean corpuscular volume was 86 fl; mean corpuscular hemoglobin was 32.1 pg; mean corpuscular hemoglobin concentration was 37.5%; and platelet count was 84 × 10^9^/l. Reticulocytes decreased to 0%. Spherocytosis was present on the peripheral blood smear. Liver function tests revealed levels of aspartate transaminase (AST) of 39 IU/l, alanine aminotransferase (ALT) of 31 IU/l, lactate dehydrogenase (LDH) of 342 IU/l, alkaline phosphatase (ALP) of 144 IU/l, γ-glutamyl transpeptidase (γ-GT) of 23 IU/l, total bilirubin of 2.9 mg/dl, and direct bilirubin of 1.0 mg/dl. Hepatitis B virus surface antigen and anti-hepatitis C virus antibody were negative. Haptoglobin concentration decreased to 2 mg/dl, and the direct antiglobulin test was negative. Phosphatidylinositol glycan deficient clone was ruled out by flow cytometry. In addition to the past history, the presence of spherocytes on the peripheral blood smear, and the presence of gallstones, the definite diagnosis of HS was made with lower fluorescence of eosin-5-maleimide (EMA)-stained red blood cells due to the decreased amount of target proteins by a flow cytometry-based test (EMA binding test) [2] and shortened the acidified glycerol lysis test (AGLT) value [3] after admission. Because of high fever, history of gallstones, and the presence of pancytopenia, empiric administration of antibiotics was initiated for possible abdominal infection. The patient received two units of packed red blood cell and showed marked clinical improvement. On the 7th hospital day, fever relapsed and gastrointestinal symptoms (abdominal discomfort and nausea) worsened. Liver function tests showed levels of AST as 492 IU/l, ALT as 320 IU/l, LDH as 517 IU/l, ALP as 351 IU/l, γ-GT as 166 IU/l, total bilirubin as 2.1 mg/dl, and direct bilirubin as 1.0 mg/dl. Computed tomography of the abdomen showed splenomegaly and gallstones, without hepatobiliary tract infection. Magnetic resonance cholangiography revealed no evidence of choledocholithiasis; blood cultures were negative. The results of anti-HPV immunoglobulin M (IgM) and immunoglobulin G (IgG) measured at admission were both positive, and HPV-B19 deoxyribonucleic acid (DNA) increased to 10^5^ copy/ml by quantitative real-time polymerase chain reaction (PCR) using the patient’s peripheral blood. Thus, HPV-B19-induced aplastic crisis was diagnosed. Because of rapid recovery of hematopoiesis and clear evidence of HPV infection, bone marrow aspiration was not performed during admission. Liver function tests returned to normal without treatment. On the 14th hospital day, the patient was discharged without any symptoms, and the hemoglobin concentration elevated to 8.9 g/dl (Figure 1).

We systematically reviewed the case reports of HPV-B19 infection that occurred in adult patients with HS and conducted a literature search using the “Pubmed” search engine. The following terms “hereditary spherocytosis” and “parvovirus B19” were used to identify the appropriate peer-reviewed, English-language papers. We collected cases of adults, defined as patients over 18 years of age, and excluded pediatric cases. In addition to the present case, we reviewed all these cases, collected clinical information described in these articles, if written, and discussed the outcome. Between 1984 and 2010, a total of 19 reports with 22 cases, including the present case were identified [4-21]. Patients’ characteristics, including those of the present case, are summarized in Table 1. Family history of HS was detected in 13 cases. Fever and liver dysfunction was documented in 18 and 4 cases, respectively. Skin manifestation was documented in only 2 cases. HPV-B19 infection was diagnosed through detection of anti-HPV B19 antibody in 12 cases, HPV-B19 DNA using PCR in 1 case, both antibody and PCR in 7 cases, and others in 2 cases.

**Table 1:** Clinical characteristics of 22 cases with human parvovirus B19 (HPV-B19) infection in adult patients with hereditary spherocytosis

**Reference**	**Age**	**Gender**	**Initial symptom**	**Family history of HS**	**Fever**	**Splenomegaly**	**Liver test abnormality**	**Skin manifestation**	**Gallstone**	**Detection of HPV-B19**
[4]	33	Female	Fever, abdominal pain, swelling of the hands, fatigue, headache, palpitations, dizziness	Yes	Yes	Yes	-	-	-	Antibody
[4]	28	Male	Lethargy, weakness, shivering, muscular pain, headache, palpitation, dizziness	Yes	-	Yes	-	-	-	Antibody
[5]	27	Male	Fever, night sweat, shivers, stiffness, headache, dry cough, dizziness	Yes	Yes	Yes	-	-	-	Antibody
[6]	27	Male	Fever, headache, pain, sweating, cough	Yes	Yes	Yes	-	-	-	Antibody
[6]	37	Female	Fever, headache, sore throat, pains, cough	-	Yes	Yes	-	-	-	Antibody
[7]	30	Female	-	-	-	-	-	-	-	Antibody
[8]	43	Female	Fever, headache, nausea, diarrhea	Yes	Yes	-	-	-	-	Antibody
[9]	34	Female	Fever, malaise, fatigue, palpitation, arthralgia, headache, dizziness	Yes	Yes	Yes	Yes	Yes	-	Antibody
[10]	34	Female	Fever, jaundice, anemia	Yes	Yes	Yes	-	-	-	Immunoelectrophoresis
[11]	18	Male	Vomiting, fever, lethargy	Yes	Yes	-	-	-	-	Antibody in situ hybridization
[12]	23	Female	Low back pain, arthralgia, fever, nausea, vomiting, diffuse abdominal pain	Yes	Yes	Yes	-	Yes	Yes	Antibody
[13]	36	Female	Fever, myalgia, malaise	-	Yes	Yes	-	-	-	Antibody, PCR
[14]	19	Male	Malaise, anorexia, night sweats	-	Yes	Yes	-	-	-	Antibody, PCR
[15]	27	Female	Arthralgia, pharyngitis, cough, nausea, vomiting, diarrhea	Yes	-	Yes	-	-	-	PCR
[16]	22	Female	Anemia, jaundice	-	-	Yes	-	-	-	Antibody
[17]	28	Male	Leg pain, fatigue	-	Yes	-	-	-	Yes	Antibody
[18]	19	Female	Fever, malaise, urinary frequency	Yes	Yes	Yes	-	-	Yes	Antibody, PCR
[18]	27	Female	Fever, malaise, splenomegaly	Yes	Yes	Yes	-	-	-	Antibody, PCR
[19]	19	Female	Nausea, vomiting, dyspnea, sever fatigue, anemia	Yes	Yes	-	Yes	-	Yes	Antibody, PCR
[20]	34	Female	Presyncope, fever, myalgia	-	Yes	Yes	-	-	-	Antibody, PCR
[21]	34	Female	Anemia, fever	-	Yes	Yes	Yes	-	Yes	Antibody
Present case	33	Female	Fever, fatigue, nausea, anemia	-	Yes	Yes	Yes	-	Yes	Antibody, PCR

A hyphen shows that there is no evidence or no description of each characteristic on the article.

HS, hereditary spherocytosis; HPV, human parvovirus; PCR, polymerase chain reaction.

## Discussion

In this study, we presented a case of HPV-B19-induced aplastic crisis in an adult patient with HS, and we performed a review of clinical features of previously published cases. The results of our review showed that all patients were young, aged 18–43 years. Retrospective studies of immunocompetent subjects infected with parvovirus B19 showed that affected patients were relatively young; in one report, the median patient age was 38 years, with 86.7% aged 26–45 years [22], while in another report [23], the median age was 32–43 years (average, 38.0 years) for males and 15–43 years (average, 34.2 years) for females. Most individuals are infected with HPV-B19 during their school years, and the percentage of those with measurable levels of B19-specific IgG increases with age. More than 70% adults have measurable levels of B19-specific IgG antibodies [24,25]. Permanent immunity from HPV may decrease the incidence of viral infection in older patients with HS.

These cases were more frequently reported in females than in males. In the epidemiologic study of HPV-B19 infection-induced aplastic crisis in 308 children with homozygous sickle cell disease, the number of infected patients did not differ between genders [26]. The analysis of HPV-B19-induced epidemic acute red cell aplasia in 26 patients, primarily in children with hereditary hemolytic anemia (including only 1 patient with HS), included 14 males and 12 females [27]. In contrast, in a retrospective study of 30 immunocompetent patients infected with parvovirus B19 in Kyoto, the male:female ratio was 4:26 (86.7% were female) [22]. Another retrospective study of 21 healthy, adult patients with HPV-B19 infection included 4 males and 17 females [23]. In humans, the genetic background probably accounts for the different patterns of HPV-induced anemia, and host genes may regulate the outcome of HPV-B19-induced aplastic crisis [28]. One may speculate that a correlation exists between genetic differences and gender gap in association with susceptibility to HPV infection, although very little is known with regard to this field.

Fever, nonspecific flu-like symptoms [1], and abdominal symptoms such as nausea or vomiting, abdominal pain, and diarrhea may occur in patients with HPV-B19-induced aplastic crisis [27]. Abdominal symptoms were also commonly observed in our review. In contrast, abnormal liver function test results during HPV-B19 infection was documented in limited cases. In pediatric patients, elevated levels of hepatic aminotransferases may accompany the fifth disease, and parvovirus infection has been associated with severe but self-limited hepatitis [29]. However, parvovirus B19 could not be implicated in a large number of adult patients with acute or chronic hepatitis [1]. The precise incidence of liver enzyme dysfunction that occurs during HPV-B19 infection in adult patients with HS is uncertain; therefore, further investigation is required. In a clinical scenario, because the development of bilirubin gallstones is a common complication of HS with chronic hemolysis, HPV infection should be considered as a part of the differential diagnosis of hepatobiliary tract infection in patients with HS since fever, abdominal pain, and liver enzyme dysfunction will also occur with such infection.

Documentation of skin manifestation was less frequent and may be considered to have less diagnostic value for HPV infection in adults. Similar to the fifth disease [1], although the skin rash is a well-known symptom, it is less characteristic in adults. In the report including 22 children with sickle cell disease or HS, no skin rash was observed during parvovirus B19-induced aplastic crisis [30]. The pathogenesis of the HPV-B19 infection-induced rash remains unclear. Because it usually coincides with the production of measurable serum antibody, it is presumed to be at least partially immune mediated [31-33]. Different immune reactions according to age may be associated with the varying incidences of skin reaction; however, the precise reason remains unclear.

HPV-B19 infects erythroid progenitor cells and inhibits erythropoiesis, leading to acute erythroblastopenia and reticulocytopenia [34]. The bone marrow in patients with transient aplastic crisis is characterized by an absence of maturing erythroid precursors and presence of giant pronormoblasts [1]. Although giant pronormoblasts are suggestive of parvovirus B19 infection, they are not diagnostic of the disease [21,24]. Because of pancytopenia accompanied with a marked decrease in reticulocytes as well as a history of HS, it was natural to believe that the patient in the present case was suffering from aplastic crisis due to viral infection; therefore, we did not perform bone marrow aspiration. Bone marrow aspiration may not be routinely required when viral infection-induced aplastic crisis is highly suspected.

## Conclusion

We report a case of aplastic crisis caused by HPV-B19 in an adult patient with HS. To the best of our knowledge, the current study is the first report that reviewed HPV-B19-induced aplastic crisis in several adult patients with HS. HPV-B19 infection-induced aplastic crisis is more common in young female patients with HS. Although fever or abdominal symptoms generally occur during HPV-B19 infection, skin manifestation may appear less commonly. It may be helpful to detect HPV-B19 infection by antibody or PCR methods to distinguish it from other infections, such as hepatobiliary infection due to gallstones, if suspected.

## Consent

Written informed consent was obtained from the patient for publication of this case report and accompanying images. A copy of the written consent is available for review by the Editor-in-Chief of this journal.

## Competing interests

The authors declare that they have no competing interests.

## Authors’ contributions

YK was responsible for the clinical management of our patient and preparation or writing of the first draft of the manuscript. YH and HK reviewed the manuscript and prepared the final draft. YI, HK and MT made substantial contributions to the acquisition and interpretation of clinical data. All authors read and approved the final manuscript.
